# Primary Angiosarcoma of the Spleen: An Oncological Enigma

**DOI:** 10.1155/2014/193036

**Published:** 2014-07-02

**Authors:** Myoteri Despoina, Dellaportas Dionysios, Ayiomamitis Georgios, Strigklis Konstantinos, Kouroumpas Efstratios, Zizi-Sermpetzoglou Adamantia

**Affiliations:** ^1^Department of Pathology, Tzaneio General Hospital, 185 36 Piraeus, Greece; ^2^2nd Department of Surgery, Aretaieion Hospital, 115 28 Athens, Greece; ^3^2nd Department of Surgery, Tzaneio General Hospital, 185 36 Piraeus, Greece

## Abstract

*Introduction*. Primary splenic angiosarcoma is an extremely unusual neoplasm originating from sinusoidal vascular endothelium. Surgical extirpation is the mainstay of treatment of this highly malignant disease. *Case Presentation*. An 82-year-old woman was admitted with left pleural effusion and a palpable left upper quadrant abdominal mass, secondary to splenomegaly by two large splenic tumors. Classic open splenectomy was performed and angiosarcoma of the spleen was the final histopathological diagnosis, which was primary since no other disease site was revealed. *Discussion*. The incidence of the disease is 0.14–0.23 cases per million, with slight male predominance. Etiology is not established and clinical presentation may confuse even experienced physicians. Imaging modalities cannot differentiate the lesion from other vascular splenic neoplasms and the correct diagnosis is mainly set after histopathological examination of the resected spleen. As with other sarcomas, surgery is the only curative approach, while chemo- and radiotherapy have poor results. Prognosis remains dismal.

## 1. Introduction

Primary splenic angiosarcoma (PSA) is among the most unusual types of malignancies [1]. It is thought to originate from splenic sinusoidal vascular endothelium and only about 200 cases have been reported so far. PSA should be included in the differential diagnosis of any patient with splenomegaly and anemia of unknown etiology. Surgical treatment with splenectomy is thought to be the only curative intervention that may result in long-term disease-free survival. Taking advantage of a case treated recently in our hospital, a short review of the current literature is provided as well.

## 2. Case Presentation

An 82-year-old woman presented to our hospital with shortness of breath and left chest pain. On primary investigation with chest X-ray a moderate left-sided pleural effusion was revealed. On history the patient complained of weight loss, fatigue, and mild afternoon fever for the last 3 months. From her past medical history, hypertension and breast cancer treated with surgery and adjuvant chemotherapy and radiation therapy 16 years ago, were significant. More specifically the patient had received six cycles of cyclophosphamide, methotrexate, and 5-fluorouracil. Physical examination revealed a palpable mass in her left upper abdominal quadrant. Laboratory findings at initial presentation revealed marked leukocytosis 44.41 × 10^9^/L, with 55% neutrophils, 42% lymphocytes, 2% monocytes, and 1% eosinophils and elevated platelet count, 551 × 10^9^/L. After admission, a thoracentesis was performed, for shortness of breath relief, and pleural fluid had findings consistent with a reactive effusion. A computed tomography (CT) scan was done, revealing splenic enlargement and two solid mass lesions measuring 10 cm and 12.5 cm in greatest diameter with ill-defined margins within the spleen. These two mass lesions were isodense to the splenic parenchyma, while they did not present contrast blush on the arterial phase and no other specific characteristic was revealed. The fundus and body of the stomach seemed compressed and dislocated anteriorly due to splenomegaly. A CT scan of the brain and thorax followed, but no other enlarged lymph nodes were found, nor indications of metastatic disease. Despite elevated white cell count and platelet count, splenomegaly was due to distinct masses identified on CT scan and no other signs indicated hematological disease.

Surgical approach in an open manner was decided for accurate diagnosis and treatment. The patient was placed on a supine position and a midline incision was preferred. Open splenectomy was performed with care not to violate the splenic capsule and not to disrupt the pancreatic tail. During the procedure, the patient received 2 units of concentrated red blood cells and 2 units of fresh frozen plasma. Her postoperative hospital course was uneventful.

**Figure 1:**
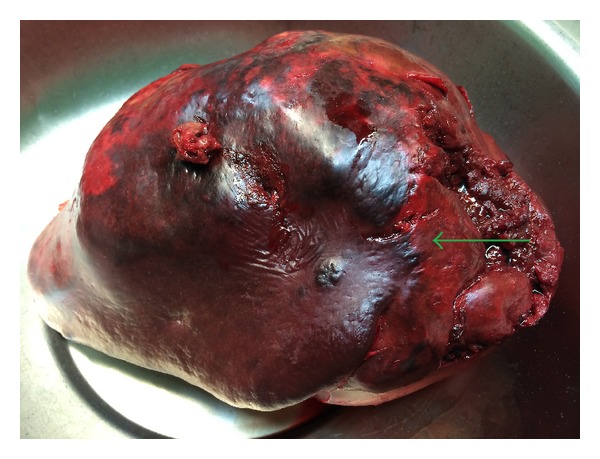
Macroscopic view of the enlarged spleen, nodular appearance (arrow).

**Figure 2:**
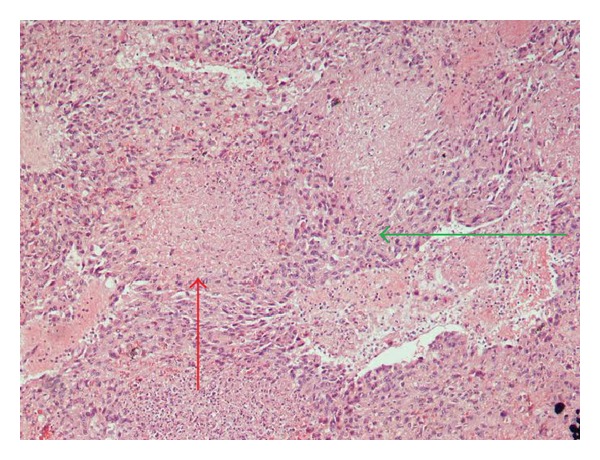
Microscopic view of splenic angiosarcoma (Hematoxylin-Eosin stain, H-E ×200). Area of atypical endothelial cells with significant irregular hyperchromatic nuclei (green arrow), area of necrosis (red arrow).

**Figure 3:**
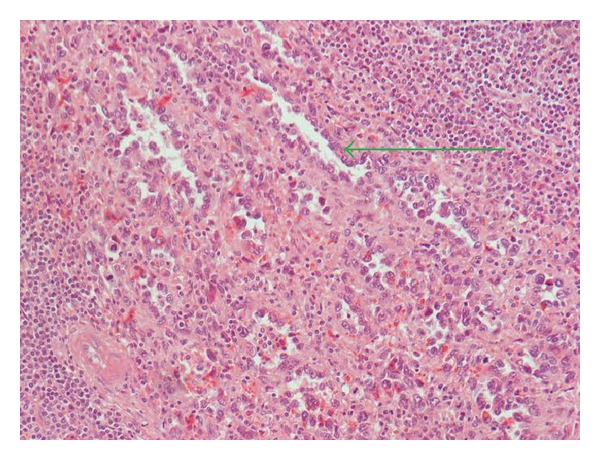
Microscopic image of the same lesion (H-E ×400), disorganized anastomosing vascular channels lined by large, atypical endothelial cells (green arrow).

**Figure 4:**
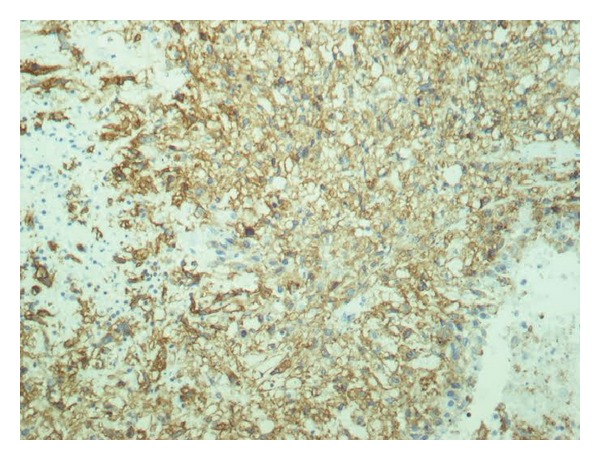
Immunohistochemical stain of angiosarcoma of the spleen (CD34 ×400).

**Figure 5:**
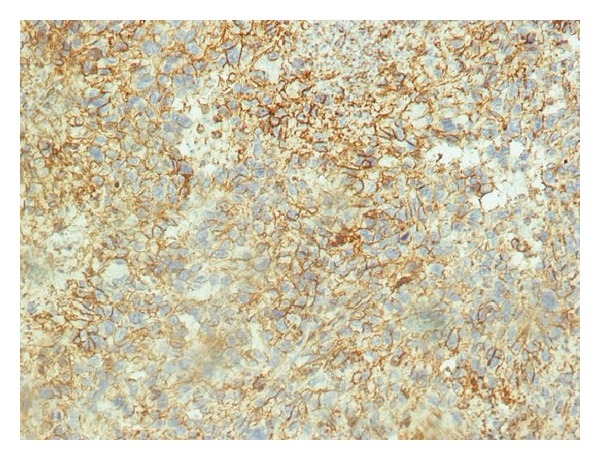
Immunohistochemical stain of angiosarcoma of the spleen (factor VIII ×400).

The spleen was 1.200 g in weight and 19 × 15 × 11 cm in size, with nodular appearance and areas of necrosis (Figure 1). The final histopathological diagnosis was angiosarcoma originating from the spleen (Figures 2 and 3). Immunohistochemical staining was positive for vimentin, CD31, CD34 (Figure 4), and factor VIII (Figure 5) and negative for CD68. The Ki67 index was less than 10%. No signs of neoplastic disease were found in an additional hilar splenule, which was found. The patient remains disease free six months later on follow-up visit.

## 3. Discussion

PSA is an aggressive malignancy originating from splenic vascular endothelium. Mesenchymal-derived elongated endothelial cells which line the spleen's spongy network of sinusoids are the primitive cells of this neoplasm. It is thought to be extremely rare, with an incidence of 0.14–0.23 cases per million, slightly predominating in men. It may arise in any age group, as cases reported are from 14 months to 89 years of age [1–3].

Pathogenesis is not accurately defined. As for every kind of angiosarcoma, thorium dioxide, vinyl chloride, and arsenic have been accused as causative ingredients; however no clear relationship between these substances and splenic angiosarcoma has been established. A few PSA patients had a history of receiving chemotherapy for lymphoma or radiation therapy for other malignancies, as our patient had prior chemo- and radiotherapy for breast cancer. In particular, alkylating agents as cyclophosphamide, which our patient received 16 years ago, are thought to be predisposing factors for other malignancies. On the other hand previous reports of PSA reported no association with chemotherapeutic agents [1]. Another attractive theory suggests that PSA may be the result of malignant transformation of other benign splenic tumors, such as hemangiomas, lymphangiomas, and hemangioendotheliomas [4–6]. Our case had no evidence of any of these factors involved.

Clinical presentation is nonspecific and may range from asymptomatic disease revealed by investigations for unrelated reasons to splenic rupture and lethal hemorrhage [7–9]. Abdominal pain in the left upper quadrant is the predominant reported symptom in more than 80% of cases [1]. Other possible nonspecific complaints include easy fatigue, anorexia, and weight loss. High temperature as an associated finding has been observed in nearly 10% of PSA patients. On physical examination not only splenomegaly is the most consistent sign [1] but also hepatomegaly and a palpable left upper quadrant mass can often be revealed. A life threatening manifestation of PSA is splenic rupture, resulting in acute abdomen and hemoperitoneum, which is reported as clinical presentation in 30% of patients [10]. Treatment is emergent splenectomy and has been proposed not to influence the final outcome and long-term survival of the patient. An extremely rare sign of the disease may be gastrointestinal bleeding, secondary to metastatic disease, which is an immerse sign [11]. Hypersplenism blood anomalies as anemia and thrombocytopenia are the most common laboratory abnormalities but thrombocytosis, leukocytosis, and elevated erythrocyte sedimentation rate are also common findings, as in our case [12].

Imaging modalities are helpful for establishing a diagnosis of splenomegaly, although lacking diagnostic accuracy for the specific disease. Ultrasound scan, computed tomography (CT), and magnetic resonance imaging (MRI) scans all display well evidence of splenomegaly. The most common findings on ultrasound are solitary or multiple, solid, and cystic mass lesions with heterogeneous echotexture. Sometimes areas of necrosis and hemorrhage are noted as cystic areas within the mass [13]. CT scan usually shows an enlarged spleen with ill-defined heterogeneously enhancing areas after contrast infusion with foci of necrosis. On acute splenic rupture, hemorrhage will appear hyperattenuated on unenhanced images, and contrast extravasation is marked after intravenous contrast infusion. Also, angiosarcomas may exhibit peripheral or heterogeneous enhancement similar to that of hepatic cavernous hemangiomas [14, 15]. According to oncologic staging for similar malignancies, areas of hypervascular metastases to the liver, lungs, bones, and lymphatic system are searched on brain, thorax, and abdominal CT scans. On MRI, both T1-weighted and T2-weighted images show ill-defined nodular lesions with increased or decreased signal intensity, related to necrosis and the presence of hemorrhage or fibrosis within the tumor, respectively [16]. Regardless of imaging modality, radiologic diagnosis of splenic angiosarcoma remains highly challenging. This is partly explained by the disease rarity and also due to overlapping characteristics with other vascular splenic tumors, such as hemangiomas, littoral cell angiomas, lymphangiomas, hemangiopericytomas, and epithelioid vascular tumors. Additionally, clinicians must include other malignant tumors in the differential diagnosis, such as lymphoma, metastatic disease, and other rare sarcomas.

In general, as with many splenic masses preoperative percutaneous biopsy is contraindicated in PSA, because of high risk of rupture and disease dissemination. Histopathologic studies can therefore only be made after operative approach and splenectomy. Specimens often weigh more than 1 kilogram. On gross examination, the spleen may be replaced entirely with malignant diffuse infiltrate. Areas of hemorrhage and necrosis are often detectable macroscopically. On the other hand, microscopically, PSA consists of disorganized anastomosing vascular channels lined by large, atypical endothelial cells with significant irregular hyperchromatic nuclei. Poorly differentiated areas have sarcomatous features, whereas well-differentiated regions appear very similar to sinuses of the spleen. Immunohistochemically, pathologists usually search for at least two vascular proliferation markers (CD31, CD34, and factor VIII) plus at least one histiocytic differentiation marker (lysozyme and/or CD68).

The only established prognostic factors are considered to be the mitotic counts and tumor size [17], for cases with no evidence of metastatic disease. Spontaneous or traumatic rupture of PSA is associated with the worst prognosis, mainly because of the immediate risk of death from hypovolemic shock and disseminated intravascular coagulopathy. Although oncologically it increases the risk of peritoneal dissemination and haematogenous spread, there is no evidence of actual difference in overall survival. According to Mark et al. histological appearance or grade is not related to outcome, because well-differentiated lesions may have a malignant potential similar to the poorly differentiated ones [18].

PSA is usually treated surgically. Splenectomy is the mainstay of treatment, because the lesion is highly refractory to adjuvant treatments with radiation and chemotherapy. PSA is an aggressive malignancy and metastases tend to occur early in disease course [19]. The reported incidence of metastases is 69% to 100% of the cases, with the most common organs affected being the liver, lungs, bones, or bone marrow, lymph nodes, gastrointestinal tract, brain, and adrenal glands [1, 2, 20]. According to a recent review for the disease, the main metastatic sites are liver (89%), lungs (78%), lymph nodes (56%), and bone (22%) [21]. Median survival of PSA is 5 months irrespective of treatment approach.

Montemayor and Caggiano indicated that splenectomy prior to rupture and dissemination of the disease is of paramount importance and median survival is 14.4 months versus 4.4 months after that incident [22]. Open approach is probably the preferred option for keeping the splenic capsule intact, or very judicious laparoscopic splenectomy, with extraction bag usage, may be applied as well in experienced hands.

In conclusion, primary angiosarcoma of the spleen is an aggressive disease that needs to be included in the differential diagnosis of splenomegaly, in cases where a distinct tumor of the spleen is revealed on imaging. Surgery is the only potentially long-term therapeutic option.
