# Erratum to “Primary Angiosarcoma of the Spleen: An Oncological Enigma”

**DOI:** 10.1155/2019/5605401

**Published:** 2019-05-08

**Authors:** Despoina Myoteri, Dionysios Dellaportas, Georgios Ayiomamitis, Konstantinos Strigklis, Efstratios Kouroumpas, Adamantia Zizi-Sermpetzoglou

**Affiliations:** ^1^Department of Pathology, Tzaneio General Hospital, 185 36 Piraeus, Greece; ^2^2nd Department of Surgery, Aretaieion Hospital, 115 28 Athens, Greece; ^3^2nd Department of Surgery, Tzaneio General Hospital, 185 36 Piraeus, Greece

In the article titled “Primary Angiosarcoma of the Spleen: An Oncological Enigma” [1], the first and last names of all the authors were reversed. The revised authors' list is shown above.
